# Molecular Detection, Epidemiology and Genetic Diversity of *Babesia microti* Infecting Wild Rodents

**DOI:** 10.1002/vms3.71196

**Published:** 2026-09-03

**Authors:** Hafiza Misbah Munir, Muhammad Naeem, Maryam Ijaz, Shakir Ullah, Muhammad Usman, Zaki Ur Rehman Nasir, Adil Khan, Khalid S. Almaary, Amir Bouallegue, Atrsaw Asrat Mengistie, Furhan Iqbal

**Affiliations:** ^1^ Institute of Zoology Bahauddin Zakariya University Multan Pakistan; ^2^ Institute of EcoHealth School of Public Health Cheeloo College of Medicine Shandong University Jinan Shandong China; ^3^ Department of Zoology Abdul Wali Khan University Mardan Pakistan; ^4^ The University of Lahore, Sargodha Campus Sargodha Pakistan; ^5^ Department of Botany and Zoology Bacha Khan University Charsadda Khyber Pakhtunkhwa Pakistan; ^6^ Department of Botany and Microbiology College of Science King Saud University Riyadh Saudi Arabia; ^7^ Laboratory of Food Oral Processing School of Food Science and Biotechnology Zhejiang Gongshang University Hangzhou China; ^8^ Department of Biology Bahir Dar University Bahir Dar Ethiopia

**Keywords:** *Babesia microti*, complete blood count, molecular prevalence, Pakistan, phylogeny, wild rodents

## Abstract

**Background:**

Rodents are considered an important natural reservoir of many zoonotic infectious agents, including parasitic Apicomplexa. To assess the potential risk of rodent‐borne parasitic protozoa in Pakistan, the present study was designed to determine the molecular prevalence and phylogenetic characteristics of *Babesia microti* in blood samples collected from *Rattus rattus* (*N* = 137), *Mus musculus* (*N* = 67) and *Rattus norvegicus* (*N* = 80).

**Methods:**

Rodents were trapped from seven districts of Pakistan between October 2023 and September 2024. PCR amplified a 238 bp fragment from the 18S rRNA gene of *B. microti* in 35 out of 284 (12%) of the screened rats and mice. DNA sequencing, BLAST and phylogenetic analysis were performed to confirm the presence and the phylogenetic similarity of the isolates. Complete blood counts were also analysed to evaluate haematological changes.

**Results:**

All three rodent species were found infected with this parasite but no significant association was observed between infection status and rodent sex or species. Statistical analysis showed that the prevalence of *B. microti* in *R. norvegicus* differed significantly between the sampling sites (*p* = 0.01), ranging from 0% in Buner, Dera Ghazi Khan and Rajanpur to 42% in Sargodha. DNA sequencing and BLAST analysis confirmed the presence of *B. microti* in rodent blood samples, with 100% sequence similarity to GenBank reference sequences. Phylogenetic analysis showed that Pakistani isolates were genetically similar and clustered with the 18S rRNA nucleotide sequences that were isolated from rodents in France, Russia and Japan. *B. microti*‐infected rodents had disturbed total white and red blood cell counts and associated parameters.

**Conclusions:**

In conclusion, we are reporting a moderate prevalence of *B. microti* in three wild Pakistani rodent species for the very first time. Further epidemiological studies are required to evaluate the potential zoonotic risk posed by *B. microti*‐infected rodents in Pakistan.

## Graphical abstract

Blood samples from 284 rodents captured across seven districts of Pakistan were screened for *Babesia microti* using 18S rRNA PCR. Overall prevalence was 12%, with infection detected in *Rattus rattus*, *Mus musculus* and *Rattus norvegicus*. Phylogenetic analysis showed close similarity with global isolates. Infected rodents exhibited altered haematological parameters, highlighting the zoonotic significance of *B. microti* in Pakistan.

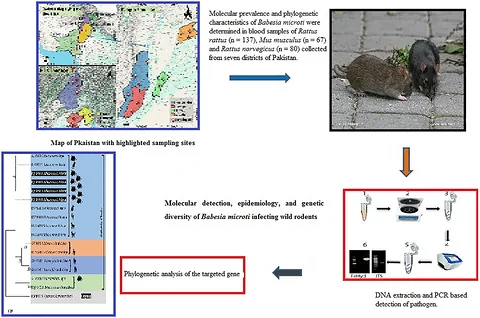

## 1 Introduction

Babesiosis is a tick‐borne disease caused by the protozoan parasites of genus *Babesia* and target the red blood cells of the host. Usually, *Babesia* spp. are transmitted to animals and humans when they are bitten by an infected tick (Kim et al. 2024). Among them, Humans are the incidental host *Babesia microti* and human babesiosis has been reported from various parts of the world including the United States, Europe and Asia (Shahzeen et al. 2025; Ramadhani et al. 2025). Additionally, *B. microti* primarily infects small rodents, especially the white‐footed mouse, which serves as its main natural reservoir. It has also been occasionally reported in various domestic animals like cats and dogs (Akram et al. 2019). This pathogen is well known for their minimum metabolic requirements to infect the red blood cells of their hosts (Joseph et al. 2012). Hard tick species *Ixodes persulcatus* in Asia, *Ixodes ricinus* in Europe and *Ixodes scapularis* in North America have been documented to be involved in the transmission of *B. microti* (Gray et al. 2002).

An enzootic cycle between ixodid ticks and small mammals, particularly rodents that serve as the principal reservoir hosts, maintains these parasites in nature, as transovarial transmission in ixodid ticks has not yet been reported (Shahzeen et al. 2025). Rodents play a critical role in the natural maintenance and amplification of *B. microti* by acting as a continuous source of infection for feeding ticks. In humans, *B. microti* can be transmitted through transfusion of contaminated blood, organ transplantation and congenital transmission from an infected mother to her foetus during pregnancy (Borggraefe et al. 2006). In most cases, the infection remains asymptomatic; however, symptoms usually appear after an incubation period of 1–4 weeks and include chills, fever, sweating, fatigue and haemolytic anaemia (Clark and Jacobson 1998; Birkenheuer et al. 2004). For human subjects, a combination of atovaquone and azithromycin is used as the preferred treatment option against babesiosis (Sari et al. 2025).

Rodents are widely distributed and have successfully adapted to diverse habitats, often living in close proximity to humans (Khan et al. 2021). They are important reservoirs of numerous zoonotic pathogens, with approximately 200 species reported to harbour over 60 zoonotic diseases caused by bacteria, viruses and parasites, including protozoans (Jahan et al. 2021; Merdana et al. 2025). From Pakistan, 43 rodent species have been documented, and most of them are different types of mice and rats. In addition, porcupines, desert gerbils and giant flying squirrels have been reported from various parts of this country, mostly acting as pests in agricultural fields (Rizwan et al. 2023). Despite having a diverse rodent fauna in Pakistan, these animals have not frequently been screened for the presence of pathogens. A quick literature review revealed a couple of reports regarding the presence of *Toxoplasma gondii* and a single report regarding the presence of *Hepatozoon* and *Lankesterella* species in local rats and mice (Ijaz et al. 2024, 2025). Rodents serve as reservoirs for numerous zoonotic pathogens that can be transmitted through diverse routes, including contamination of food or water with urine or faeces, predation on infected rodents or transmission by ectoparasites. These transmission pathways highlight the importance of screening rodent populations for zoonotic pathogens (Rabiee et al. 2018). Hence, this investigation was designed to report the prevalence of *B. microti* in the blood samples of wild rodent species that were trapped from seven districts in Pakistan. We are also reporting the risk factors associated with *B. microti* infection in rats and mice, as well as the effects of this pathogen on the complete blood count of the screened rodents.

## 2 Materials and Methods

### 2.1 Study Area and Subjects

**FIGURE 1:**
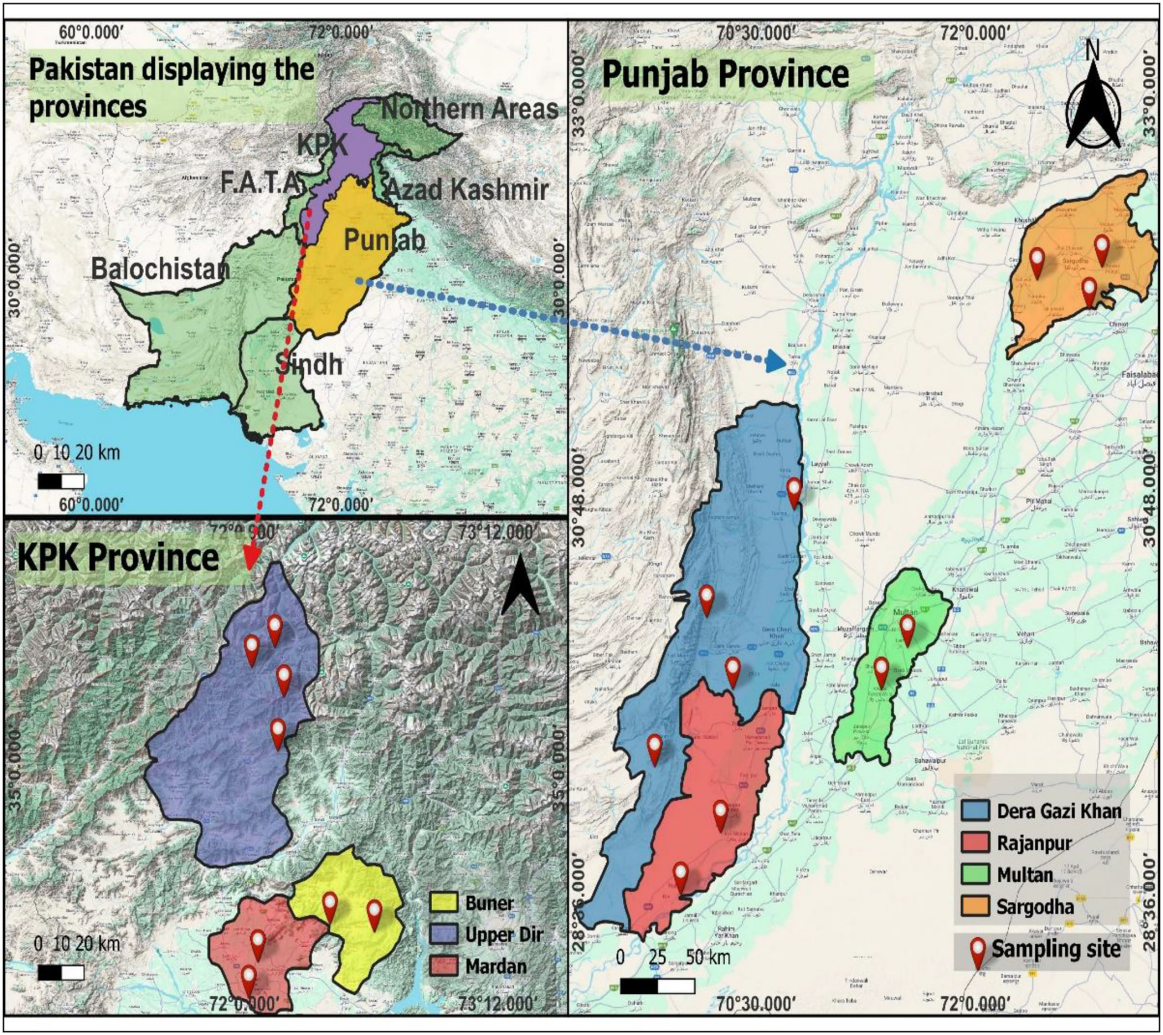
Map of Pakistan showing the location of Punjab (yellow) and Khyber Pakhtunkhwa (purple) provinces. Magnified maps of the two provinces present the districts from where the rodent blood samples were collected.

During the current investigation, the prevalence of *B. microti* was determined among the wild rodents that were trapped from Punjab and Khyber Pakhtunkhwa (KPK) provinces in Pakistan during October 2023 till September 2024 (Figure 1). All the animal handling procedures and experiment protocols were reviewed and approved by the ethical review committee of Bahauddin Zakariya University, Multan, Pakistan (BZU/Ethics/Zool‐42‐2023). Study was conducted in accordance with ARRIVE guidelines.

The required rodent sample size was calculated using the Thrusfield formula for prevalence studies, assuming an expected prevalence of 10%, a 95% confidence level and a desired precision of 5%: *n* = (*Z* ^2^ × Pexp × (1 − Pexp))/*d* ^2^ (Thrusfield 2018). This yielded a minimum required sample size of 138 rodents. However, 284 rodents were trapped to improve the precision and reliability of the prevalence estimates. Rodent trapping was conducted opportunistically at different sampling sites. Live rodent traps with fruit, sweets and biscuits as bait, as well as glue traps, were used to trap rodents from those sites where rodents were abundant and active, including agricultural fields, houses, storage sites and from commercial shops in the sampling areas. The number of traps deployed varied among sites depending on location characteristics, accessibility and the field convenience, and therefore no fixed number of traps was maintained per site. Traps were not set daily throughout the study period; however, whenever deployed, they were checked at intervals of a few hours to ensure timely retrieval of captured rodents. The captured rodents were carefully handled for blood collection when they were trapped alive. Standard taxonomic keys were used to identify the species of the trapped rodents (Smithers 1986).

### 2.2 Data and Blood Sample Collection

A screening sheet was filled to collect information about each animal in order to calculate the risk factors (sampling site, rodent species and animal sex) associated with the prevalence of *B. microti* in trapped rodents. Body of each animal was placed on surgical sheet and combed from tail towards head with a fine comb in order to collect the dislodged ectoparasites. The trapped rodents were euthanized under deep isoflurane anaesthesia. Blood samples were collected from each animal from direct cardiac puncture under isoflurane inhalation (Zhu et al. 2010). Blood samples were collected in labelled tubes containing 0.5 M EDTA as an anticoagulant. Complete blood count was performed on the same day, when the blood was sampled, whereas the remaining blood was frozen until the molecular analysis.

### 2.3 Complete Blood Count Analysis

Haematological analyser (Mythic 18 Vet, Orphee, Switzerland) was used to determine the complete blood count parameters of rodents to be compared between parasites infected and uninfected animals, where possible.

### 2.4 DNA Extraction

Genomic DNA was extracted from the rodent blood by using commercial DNA purification kit (Promega, Madison, WI, USA) following the manufacturer's instructions.

### 2.5 Molecular Detection by PCR

The extracted DNA samples were analysed for the presence of *B. microti* (target was 18S rRNA gene) by using the species‐specific primers (Forward 5′CTTAGTATAAGCTTTTATACAGC‐3′ and Reverse 5′ATAGGTCAGAAACTTGAATGATACA 3′) following Persing et al. (1992). For the molecular detection of *B. microti*, a reaction mixture of 50 µL was prepared containing 1.5 mM MgCl_2_, 10× PCR buffer, 5 µL of template DNA, 0.2 mM deoxyribonucleotide triphosphates, 2 U of Taq DNA Polymerase (Parstous, Mashhad, Iran) and 0.5 mM of each primer (Persing et al. 1992). The thermo‐profile consisted of an initial denaturing step of 5 min at 94°C that was followed by 30 cycles of denaturing for 1 min at 94°C, annealing for 1 min at 55°C and elongation for 2 min at 72°C. There was a final extension for 7 min at 72°C following Persing et al. (1992). In each PCR run, distilled water was included as a negative control, whereas DNA extracted from a previously confirmed *B. microti*‐positive dog blood sample from our laboratory (Shahzeen et al. 2025) served as the positive control.

### 2.6 DNA Sequencing and Phylogenetic Analysis of the 18S rRNA Gene of *Babesia microti*

Amplified partial 18S rRNA gene fragments of *B. microti* were confirmed by bidirectional DNA sequencing using the same primers employed for PCR amplification. The obtained sequences showed high similarity to reference *B. microti* sequences, confirming the specificity of the assay. Two representative sequences from *Rattus norvegicus* and one each from *R. rattus* and *Mus musculus* were subsequently included in the phylogenetic analysis. These sequences were submitted to GenBank and were assigned with accession numbers PQ538588, PQ538589, PQ538590 and PQ538591. The generated sequences were screened in FinchTV (version 1.4.0) to remove the low‐quality nucleotides at both ends of the sequence. The remaining clean sequences were saved in FASTA format and used in BLAST analysis. Similar sequences were downloaded from the BLAST output to be used in the phylogenetic analysis. These sequences were aligned by using the ClustalW multiple sequence alignment tool. The evolutionary tree was inferred by using the Maximum Likelihood method and Kimura 2‐parameter model. The tree with the highest log likelihood (−484.01) is shown. The percentage of trees in which the associated taxa clustered together is shown next to the branches. Initial tree(s) for the heuristic search were obtained automatically by applying Neighbor‐Joining and BioNJ algorithms to a matrix of pairwise distances estimated using the maximum composite likelihood (MCL) approach, and then selecting the topology with superior log likelihood value. A discrete gamma distribution was used to model evolutionary rate differences among sites (5 categories (+*G*, parameter = 0.4037)). The tree is drawn to scale, with branch lengths measured in the number of substitutions per site. This analysis involved 17 nucleotide sequences. There were a total of 195 positions in the final dataset. Evolutionary analyses were conducted in MEGA X (https://www.megasoftware.net/). The final version of the inferred tree was generated by using the iTOL server. The 18S rRNA gene of *Cytauxzoon brasiliensis* (PQ686812) served as an outgroup for this study.

### 2.7 Statistical Analysis

Statistical package Minitab (Minitab, PA, USA) was used for the data analysis. Significance level was set at *p* ≤ 0.05. Data were presented as mean ± standard error of mean for complete blood count parameters and were compared between infected and uninfected rodents through a sample *t*‐test. Prevalence of *B. microti* between rodent species was compared through a chi‐square (χ^2^) test. The association between sampling sites and screened pathogens was assessed using binary logistic regression. In this analysis, Rajanpur served as the reference district. Odds ratios (ORs) and 95% confidence intervals (CIs) were calculated to compare infection risk. Fisher's exact test was calculated to report the association of the pathogen with rodent sex.

## 3 Results

### 3.1 Taxonomic Identification of the Trapped Rodents

A total of 284 wild rodents were trapped from two provinces during the present investigation. From Punjab, 169 (from four districts) and 115 rodents were trapped from KPK (from three districts). The rodents included in this study were 54% male (153/284) and 46% female (131/284). From Punjab, 40, 48, 49 and 32 rodents were trapped from Rajanpur, Dera Ghazi Khan, Multan and Sargodha districts, respectively. However, 54 rodents from Upper Dir, 34 from Mardan and 27 rodents were captured from the Buner district in KPK. These rodents belonged to three species, including *M. musculus* (house mouse, *N* = 67), *R. rattus* (black rat, *N* = 137) and *R. norvegicus* (brown rat, *N* = 80). All rodents were thoroughly examined for the presence of ticks; however, no ticks were detected throughout the study period.

### 3.2 Prevalence of *Babesia microti* Among the Trapped Rodents

PCR amplified a 238‐base‐pair amplicon specific for the 18S rRNA gene of *B. microti* in 35 out of 284 (12%) blood samples of three wild rodent species that were collected from two provinces during the present study (Table 1).

**TABLE 1:** Overall comparison of *Babesia microti* prevalence among the three rodent species captured from two provinces in Pakistan during the present investigation.

Rodent species	*Babesia microti* +ve	*Babesia microti* −ve	*p* value
*Mus musculus*	4/67 (6%)	63/67 (94%)	
*Rattus rattus*	18/137 (13%)	119/137 (87%)	0.1
*Rattus norvegicus*	13/80 (16%)	67/80 (84%)	
**Grand total**	**35/284 (12%)**	**249/284 (88%)**	

*Note*: % Prevalence is given in parentheses. *p* value represents the output of the chi‐square (χ^2^) test. *p* > 0.05 = non‐significant.

### 3.3 Phylogenetic Analysis Based on 18S rRNA Gene of *Babesia microti* Among Pakistani Wild Rodents

**FIGURE 2:**
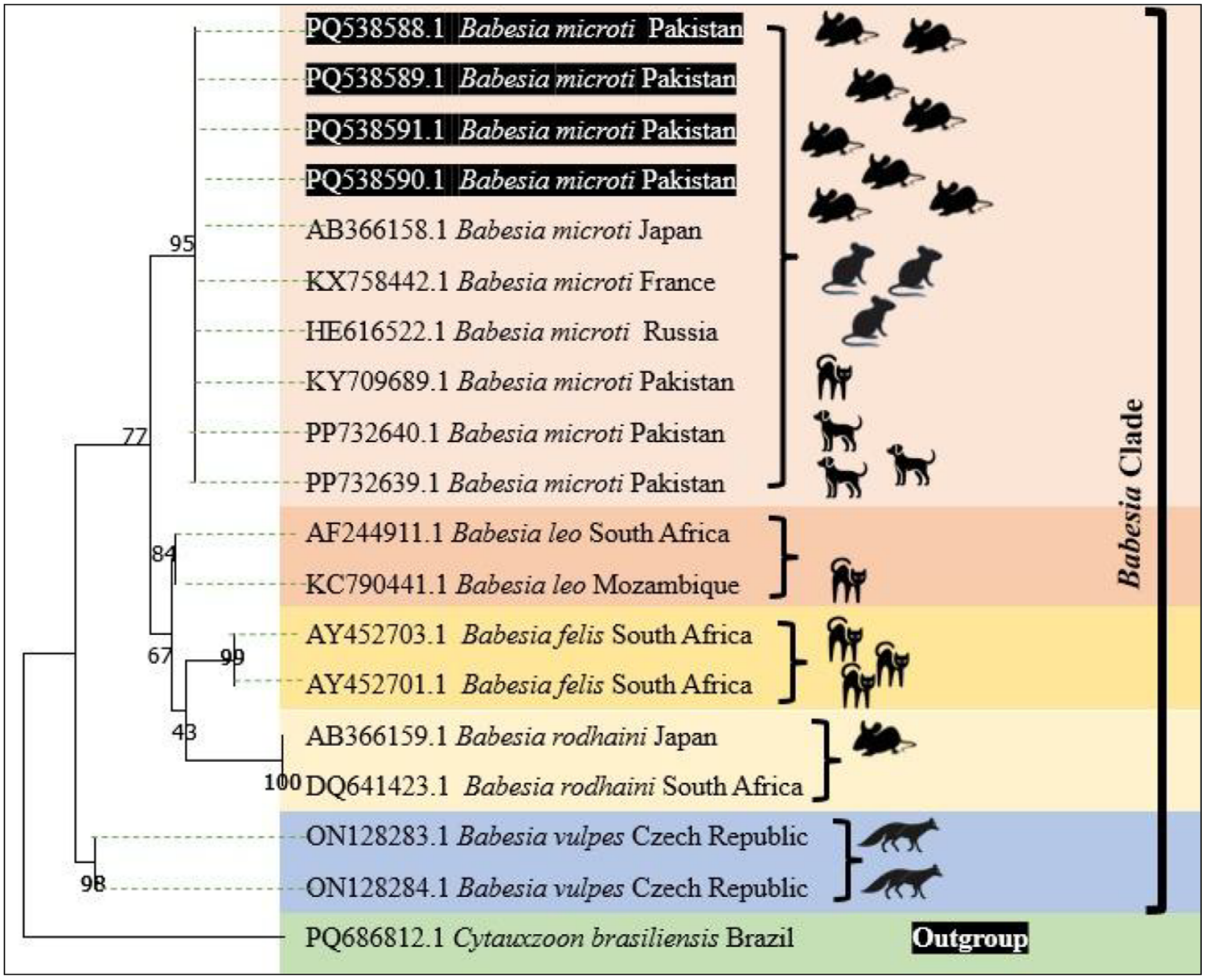
Phylogenetic tree of *Babesia microti* based on the partial 18S rRNA gene sequences available in GenBank. The four 18S rRNA gene sequences (PQ538588, PQ538589, PQ538590 and PQ538591) generated in this study are highlighted in black.

Analysis of the partial 18S rRNA gene sequence of *B. microti* revealed that the sequences generated in this investigation (accession numbers PQ538588–PQ538591) were genetically similar, as they resided in the same cluster (Figure 2). Our 18S rRNA gene sequences showed 100% identity with the *B. microti* sequences detected in dog and cat blood samples from Pakistan (accession numbers KY709689, PP732639 and PP732640), 99.49% identity with *B. microti* isolated from the blood of *M. musculus* in Japan (accession number AB366158), 99.49% identity with *B. microti* isolated from the spleen of *M. musculus* from France (accession number KX758442), 99.49% identity of our sequences was also observed with *B. microti* isolated from the blood of *Myodes rufocanus* (rodent) (accession number HE616522) in Russia. In comparison, our sequences were distinct from 18S rRNA sequences of *Babesia leo, Babesia felis, Babesia vulpes* and *Babesia rodhaini* detected in felines and rodents from South Africa, Mozambique, Czech Republic and Japan, respectively (Figure 2).

### 3.4 Risk Factor Analysis

Three wild rodent species were identified during the present investigation. χ^2^ test results indicated that the prevalence of *B. microti* varied non‐significantly (*p* = 0.1) when compared among the captured rodent species (Table 1).

Binary logistic regression revealed no significant association between sampling district and *B. microti* infection in *M. musculus*, *R. rattus* or *R. norvegicus* (all *p* > 0.05). Although the observed prevalence varied among districts, with the highest prevalence recorded in Buner (25%) for *M. musculus*, Mardan (28%) for *R. rattus* and Sargodha (42%) for *R. norvegicus*, these differences were not statistically significant (Table 2).

**TABLE 2:** Overall comparison of *Babesia microti* prevalence in a particular rodent species that was captured from seven sampling districts during the present investigation.

Sampling districts	Infected *Mus musculus*	OR (vs. Rajanpur (95% CI))	*p* value	Infected *Rattus rattus*	OR (vs. Rajanpur (95% CI))	*p* value	Infected *Rattus norvegicus*	OR (vs. Rajanpur (95% CI))	*p* value
Rajanpur*N* = 40	0/34 (0%)	1.0 (Ref)	—	0/2 (0%)	1.0 (Ref)	—	0/4 (0%)	1.0 (Ref)	—
Dera Ghazi Khan *N* = 48	—	—	—	2/30 (7%)	0 (0.0000–1.41207E+191)	0.966	0/18 (0%)	1 (0.0000–6.28340E+152)	1
Multan *N* = 49	0/7 (0%)	1 (0.0000–5.36897E+114)	1	2/26 (8%)	0 (0.0000–1.21038E+191	0.965	2/16 (13%)	0 (0.0000–3.70017E+133)	0.947
Sargodha *N* = 32	0/1 (0%)	1 (0.0000–2.87019E+280)	**1**	3/12 (25%)	0 (0.0000, 3.02469E+190)	0.96	8/19 (42%)	0 (0.0000–7.81024E+132)	0.94
Upper Dir *N* = 54	1/11 (9%)	0 (0.0000–9.05885E+42)	0.854	4/33 (12%)	0 (0.0000, 7.30467E+190)	0.963	1/10 (10%)	0 (0.0000–5.13985E+133)	0.949
Mardan *N* = 34	1/6 (17%)	0 (0.0000–4.53740E+42)	0.844	5/18 (28%)	0 (0.0000, 2.61952E+190)	0.96	2/10 (20%)	0 (0.0000–2.27769E+133)	0.945
Buner *N* = 27	2/8 (25%)	0 (0.0000–2.69701E+42)	0.837	2/16 (13%)	0 (0.0000, 7.06143E+190)	0.963	0/3 (0%)	0 (0.0000–1.32144E+211)	1

*Note*: % Prevalence of pathogen is given in parenthesis. *p* value represents the results of significance for binary logistic regression calculated for studied parameter. *p* > 0.05 = non‐significant; *p* < 0.01 = significant (**).

Abbreviations: CI = 95% confidence intervals; OR = odds ratios.

Fisher's exact test results indicated that *B. microti* infection was not limited to a specific sex of *M. musculus* (*p* = 1)*, R. rattus* (*p* = 0.3) and *R. norvegicus* (*p* = 0.7) trapped from seven districts during the present study (Table 3).

**TABLE 3:** Overall association of animal sex with the prevalence of *Babesia microti* in a particular wild rodent species captured from seven districts during the present study.

Rodent species	Parameters		*Babesia microti* positive samples	*Babesia microti* negative samples	*p* value
*Mus musculus*	Sex	Male	0/38 (5%)	36/38 (95%)	1
		Female	2/29 (7%)	27/29 (93%)	
*Rattus rattus*	Sex	Male	12/74 (16%)	62/74 (84%)	0.3
		Female	6/63 (10%)	57/63 (90%)	
*Rattus norvegicus*	Sex	Male	6/41 (15%)	35/41 (85%)	0.7
		Female	7/39 (18%)	32/39 (82%)	

*Note*: Percentage prevalence is presented in parentheses. *p* value represents the outcome of Fisher's exact test. *p* > 0.05 = non‐significant.

### 3.5 Rodent's Body Weight Analysis

Analysis of two‐sample *t*‐test revealed that body weight of *B. microti*‐infected *M. musculus* was significantly reduced than the uninfected mice that were trapped from seven districts during the present investigation (*p* = 0.02) (Figure S1). Although the body weight of parasite‐infected *R. rattus* (*p* = 0.4) and *R. norvegicus* (*p* = 0.3) was higher than that of uninfected rodents, the difference was not statistically significant (Figure S1).

### 3.6 Complete Blood Count Analysis

For *R. rattus*, total white blood cells (*p* = 0.05), lymphocytes (*p* = 0.001) and red blood cell count (*p* = 0.02) were significantly reduced in *B. microti‐*infected *R. rattus* than those black rats that were not found infected with the parasite (Table 4). For *R. norvegicus*, lymphocytes (*p* = 0.003) and mean corpuscular volume (*p* = 0.001) were significantly decreased, whereas mean corpuscular haemoglobin concentration (*p* < 0.0001) was significantly elevated in *R. norvegicus* that were infected with *B. microti* than the uninfected rodents (Table 4).

**TABLE 4:** Overall comparison of studied complete blood count parameters between *Babesia microti‐infected* and uninfected wild rodent species captured from various sampling sites in Pakistan.

Studied parameters	*Babesia microti* positive *Rattus norvegicus* (*n* = 13)	*Babesia microti* negative *Rattus norvegicus* (*n* = 67)	*p* value	*Babesia microti* positive *Rattus rattus* (*n* = 18)	*Babesia microti* negative *Rattus rattus* (*n* = 119)	*p* value
White blood cells ×10^3^/µL	5.10 ± 0.83	6.9 ± 1.3	0.2	4.6 ± 1.6	13.9 ± 4.4	0.05*
Lymphocytes ×10^3^/µL	3.12 ± 0.45	15.7 ± 3.9	0.003**	3.19 ± 0.81	7.88 ± 0.93	0.001***
Red blood cells ×10^6^/µL	4.89 ± 0.44	4.78 ± 0.34	0.853	5.41 ± 0.44	6.58 ± 2.8	0.02*
Haemoglobin (g/dL)	10.04 ± 0.91	10.01 ± 0.69	0.983	11.29 ± 0.61	9.84 ± 0.38	0.07
Haematocrite (%) HCT	26.3 ± 2.2	29.9 ± 2.1	0.243	34.46 ± 2.5	33.6 ± 1.9	0.8
Mean corpuscular volume (µm^3^) MCV	53.41 ± 1.9	67.1 ± 3.5	0.001***	64.2 ± 1.4	62.1 ± 2.1	0.4
Mean cell haemoglobin (pg) MCH	20.57 ± 0.24	20.0 ± 0.51	0.3	20.91 ± 0.75	22.69 ± 0.61	0.08
Mean corpuscular haemoglobin concentration (g/dL) MCHC	38.06 ± 0.69	32.88 ± 0.81	*p* < 0.0001***	32.83 ± 1.1	31.63 ± 1.0	0.4
Platelets ×10^3^/µL	340 ± 76	243 ± 39	0.2	502 ± 129	346 ± 34	0.3

*Note: n* represents the number of rodents included in each comparison group (infected or uninfected). All values are expressed as mean ± standard error of mean. *p* value indicates the results of *t*‐tests calculated for each parameter. *p* > 0.05 = non‐significant; *p* < 0.05 = least significant (*); *p* < 0.01 = significant (**); *p* < 0.001 = highly significant (***).

Statistical analysis was not possible for *M. musculus* as complete blood count data were not available for *B. microti‐*infected and uninfected *M. musculus* captured from various sampling districts during the present investigation.

## 4 Discussion

Rodents play an important role in maintaining and the transmitting zoonotic parasites to humans and animals as they are living in their nearby areas (Selmi et al. 2021). *B. microti* is known to infect the rodents, but this important zoonotic pathogen has never been investigated in Pakistan. Hence, here we are providing the first report regarding the presence of this apicomplexan parasite in three wild rodent species of Pakistan.

Screening of Pakistani wild rodent species revealed that overall, 12% of the trapped animals were infected with *B. microti*. Both mouse (*M. musculus*) and rat species (*R. rattus* and *R. norvegicus*) captured in this study from two provinces in Pakistan had this parasitic infection (Table 1). Although this parasite is known to cause human babesiosis, there are few reports available in the literature reporting *B. microti* infection among rodents. Santodomingo et al. (2022) found that 22% (15/102) of the screened small rodents (belonging to 10 species) captured in Chile were *B. microti‐*infected. Parasitic infection was 10.7% (27/252) in liver and spleen samples of rodents from China (Liu et al. 2024), 10% (33/331) in rodents from South Korea (Kim et al. 2024) 5.8% (31/536) in screened rodents from four provinces in Turkey (Usluca et al. 2019), 5.6% (81/1439) in rodents from Mainland South East Asia (Karnchanabanthoeng et al. 2018), 4.3% (72/1672) in wild rodents from Yunnan province in China (Chen et al. 2017), 2.8% (33/1180) in eight species of rodent trapped in Lithuania (Mardosaitė‐Busaitienė et al. 2021), 1.4% (8/314) in wood mice and bank voles in Ireland (Zintl et al. 2023) and 0.6% (3/498) in spleen samples of *Microtus socialis* in Turkey (Guven et al. 2022). *B. microti* was not detected in *R. rattus* from Italy (Zanet et al. 2023) and *R. rattus* that were trapped from eight European countries (Obiegala et al. 2019). In all these investigations, 18S rRNA gene of *B. microti* was targeted for its detection, indicating that it is a conserved, widely validated molecular marker suitable for sensitive detection and preliminary phylogenetic characterization, although its resolution for detailed genotypic differentiation remains limited (Ali et al. 2024). These variations in the *B. microti* prevalence are likely due to the different climatic and geographical conditions of the studied areas, sampling season, variation in age and host immunity and are also due to the variations in tick density among the sampling sites (Kim et al. 2024).

Phylogenetic trees are constructed to reflect evolutionary relationships among organisms, and for this purpose, conserved genes of these organisms are targeted. For the pathogens, 18S rRNA genes are among the preferred genes that are used to reconstruct their evolutionary histories due to their conserved nature and slow evolutionary rates (Wu et al. 2015). The phylogenetic diversity of *B. microti* has been documented in dogs (Ali et al. 2024), but no information is available to date regarding the phylogenetic diversity of this pathogen among Pakistani wild rodents. The phylogenetic tree revealed that the 18S rRNA sequences amplified during this investigation were highly conserved and closely resembled the *B. microti* sequences that were isolated from cats and dogs in Pakistan (Akram et al. 2019; Ali et al. 2024), rodents in Japan (Takabatake et al. 2009) and Russia (Samokhvalov et al. 2010), and small mammals in France (Jouglin et al. 2017). Pakistani isolates were distinct from the 18S rRNA sequences of three *Babesia* species that were isolated from cats and rodents. We used *C. brasiliensis* as the out group because it is genetically distinct and formed a separate clade from all other *Babesia* species that were used in this phylogenetic analysis, making it a suitable candidate for rooting the phylogenetic tree (Figure 2). These limited data demand that the presence of *B. microti* should be explored in wild rodents from Pakistan as well as worldwide in order to understand the phylogenetic diversity of this parasite that will lead to its effective control.

Risk factor analysis showed that *Rattus* species had a higher prevalence of *B. microti* infection than mice. Notably, all *M. musculus* infected with *B. microti* were trapped in KPK, whereas none of the mice from Punjab tested positive. In contrast, both *R. rattus* and *R. norvegicus* were found infected in both provinces (Tables 1 and 2). The observed difference in infection rates between rodent species may be explained by several factors. Rats, owing to their larger body size, may host more ticks; however, mice are generally recognized as more competent reservoirs for tick‐borne pathogens, including *B. microti* (Hersh et al. 2012). Thus, body size alone does not determine infection risk. Instead, a combination of factors such as body size, host behaviour and habitat, movement range, tick host preference and reservoir competence collectively shape the prevalence of *B. microti* across rodent species (Allsopp et al. 1994). These findings highlight the need for region‐specific surveillance of rodent populations in Pakistan, as both species and geography appear to influence the risk of *B. microti* transmission to humans and animals. Our results indicated that *B. microti* infection was not limited to a particular rodent species, sampling district or sex during the present investigation (Tables 2 and 3). Our results are in agreement with Liu et al. (2024) as they found that *B. microti* infection rates varied non‐significantly between rodent sex as well as in the tissues that were screened for parasite detection. Contrary to our findings, they found that the parasite prevalence varied significantly when compared between different rodent species, habitats or sampling sites in China. Mardosaitė‐Busaitienė et al. (2021) and Kim et al. (2024) also found that the parasite prevalence varied significantly between the animals trapped from various habitats in Lithuania and South Korea, respectively. Chen et al. (2017) found that rodents from forests and shrublands in China had significantly higher *B. microti* infection rates than those trapped from rain‐fed cropland, orchard and irrigated cropland. Usluca et al. (2019) also documented that *B. microti* prevalence significantly varied between the screened rodent species from Turkey, and the highest prevalence was observed in *Myodes glareolus*. Similar to our observation, Zintl et al. (2023) and Guven et al. (2022) found that the parasite prevalence was not limited to small rodents trapped from a particular sampling site in Ireland and Turkey, respectively. Karnchanabanthoeng et al. (2018) documented that the infection by *Babesia* spp. was more likely to be present in rodents settled near forested areas, which may represent risky places for the spread of *Babesia* species to humans. During the present study, we observed that *B. microti*‐infected house mice (*M. musculus*) had significantly reduced body weight as compared to mice in which this parasite was not detected (Figure S1). Although *B. microti* positivity was associated with lower body weight in *M. musculus*, the observational design of this study does not allow a causal relationship to be inferred. Other factors, such as age, nutritional status, reproductive condition, concurrent infections or environmental conditions, may also have contributed to the observed differences in body weight (Usluca et al. 2019). Our observation is in line with Yu‐Chun et al. (2018) as they had also found significantly lower body weights of experimentally infected BALB/c mice on seventh day post *B. microti* infection as compared to the uninfected mice. No ticks were observed on the trapped rodents. This may be attributed to the temporary attachment of ticks during blood feeding, after which they detach to continue their development in the environment (Ijaz et al. 2025). In addition, seasonal variation in tick activity, host grooming behaviour and the timing of rodent trapping may have reduced the likelihood of detecting attached ticks (Rabiee et al. 2018).

Complete blood count analysis is a routine test in pathology labs to report the effect of blood‐borne parasites in rodents. Although the blood collection from rodents, especially from mice, is not convenient due to their small body size and blood volumes they contain (Liu et al. 2022). It has been an established fact that following the bite of an infected *Ixodes* tick, *B. microti* sporozoites are transmitted into the host, where they invade erythrocytes and undergo asexual replication. The repeated rupture of infected red blood cells during merozoite release results in haemolytic anaemia and the associated clinical manifestations (Westblade et al. 2017). Our results are in agreement with these universal findings, as we had also observed a significant reduction in total red and white blood cell count and associated parameters of both rat species that were screened during the present investigation (Table 4). Multiple haematological parameters were analysed using separate Student *t‐*tests without adjustment for multiple comparisons; there is an increased risk of Type I error. Therefore, statistically significant findings should be interpreted with caution and validated in future studies with larger sample sizes. A few studies have reported the effect of *B. microti* infection on the blood cells or reported their presence in red blood cells under laboratory conditions (Clark and Jacobson 1998), but there are no such data available in the literature from wild rodents. In a similar study, Yu‐Chun et al. (2018) injected *B. microti* into 6‐week‐old BALB/c mice, and they observed a decline in red blood cells, haemoglobin and platelet count in infected mice. This decrease was inversely proportional to the parasitemia, but these parameters were returned to normal levels during the chronic infection phase. Hence, the data generated in this study adds to the existing knowledge regarding the effect of this protozoan parasite on the blood cell count and associated parameters of three Pakistani wild rodent species.

A limitation of this study is that rodent trapping was opportunistic, and the number of traps deployed varied among sampling sites due to differences in site accessibility and field conditions. Consequently, the sampled rodents may not fully represent the rodent populations at each location, and the findings should be interpreted with this potential sampling bias in mind. Another limitation of this study is that potential risk factors, including rodent age, body condition, habitat type and seasonal variation, were not evaluated. Future studies incorporating these variables would provide a more comprehensive understanding of the epidemiology of *B. microti*. Ticks are the primary vectors of *B. microti* in animals and humans. No ticks were detected on the trapped rodents, likely because ticks attach only temporarily for blood feeding before detaching to continue their development in the environment (Gray et al. 2002). Future studies should investigate rodent‐associated ticks by using morphological and molecular approaches and to screen them for *B. microti* and other tick‐borne pathogens to better understand transmission dynamics. Although no human babesiosis cases have been reported in Pakistan, undiagnosed infections may occur due to limited awareness, inadequate diagnostic facilities and underrecognition of tick‐borne diseases, particularly in rural areas. The possibility of undiagnosed human infections warrants future investigation. A major limitation of this study was the absence of blood smear examination. Because samples were collected under field conditions, immediate preparation of high‐quality blood smears was not feasible. Consequently, parasite detection relied exclusively on sensitive molecular methods. Although PCR provides high sensitivity for detecting *B. microti* DNA, it does not allow morphological confirmation of the parasite or estimation of parasitemia, making it impossible to distinguish between low‐level and heavy infections or to assess parasite burden. Therefore, the prevalence reported in this study should be interpreted as molecular detection rather than confirmation of active parasitemia. Future studies should integrate molecular diagnostics with microscopic examination to enable morphological verification, parasitemia assessment and a more comprehensive evaluation of infection status.

In conclusion, we are reporting a moderate *B. microti* infection in three Pakistani wild rodent species, and *R. norvegicus* had the highest rate of infection. Parasite prevalence did not vary between the rodent species. *B. microti* infection disturbed the red and white blood cells and associated parameters of infected rodents. Further epidemiological studies are required to evaluate the potential zoonotic risk posed by *B. microti*‐infected rodents in Pakistan.

## Author Contributions

Conceptualization, original draft writing, reviewing and editing: Hafiza Misbah Munir, Muhammad Naeem, Maryam Ijaz and Shakir Ullah. Formal analysis, investigations, funding acquisition, reviewing and editing: Muhammad Usman, Zaki Ur Rehman Nasir, Adil Khan and Khalid S. Almaary. Resources, data validation, data curation and supervision: Amir Bouallegue, Atrsaw Asrat Mengistie and Furhan Iqbal.

## Funding

This work is financially supported by the Ongoing Research Funding Program (ORF‐2026‐189), King Saud University, Riyadh, Saudi Arabia

## Ethics Statement

Animal handling and experiment protocols were reviewed and approved by the ethical review committee of Bahauddin Zakariya University, Multan, Pakistan (BZU/Ethics/Zool‐42‐2023).

## Consent

The authors have nothing to report.

## Conflicts of Interest

The authors declare no conflicts of interest.

## ARRIVE Guidelines

The experimentation was conducted in accordance with applicable laws and ARRIVE guidelines.

## Supporting information




**Supporting File: 1** vms371196‐sup‐0001‐SuppMat.docx

**Supporting File: 2** vms371196‐sup‐0002‐SuppMat.docx

## Data Availability

The datasets generated and/or analysed during the current study are available in the GenBank repository, with Accession numbers PQ538588, PQ538589, PQ538590 and PQ538591 (https://www.ncbi.nlm.nih.gov/nuccore/ PQ538588, https://www.ncbi.nlm.nih.gov/nuccore/ PQ538589, https://www.ncbi.nlm.nih.gov/nuccore/ PQ538590 and https://www.ncbi.nlm.nih.gov/nuccore/ PQ538591).
