# Supplementary material for: Molecular Detection, Epidemiology and Genetic Diversity of Babesia microti Infecting Wild Rodents
Source: Vet Med Sci. 2026 Sep 3;12(5):e71196. doi: 10.1002/vms3.71196 (PMC13542696; doi:10.1002/vms3.71196)

Molecular detection, epidemiology, and genetic diversity of *Babesia microti* infecting wild rodents

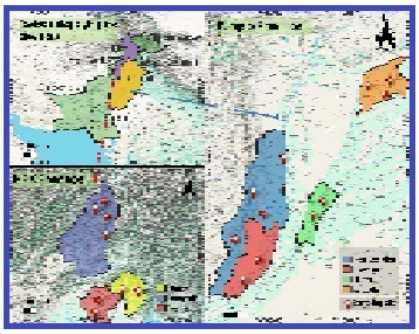

Map of Pkaistan with highlighted sampling sites

Molecular prevalence and phylogenetic characteristics of *Babesia microti* were determined in blood samples of *Rattus rattus* (n = 137), *Mus musculus* (n = 67) and *Rattus norvegicus* (n = 80) collected from seven districts of Pakistan.

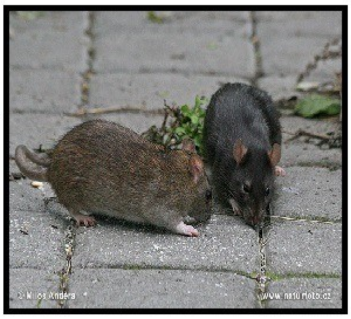

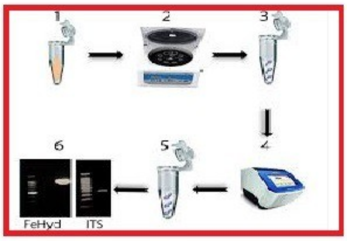

DNA extraction and PCR based detection of pathogen.

Phylogenetic analysis of the targeted gene

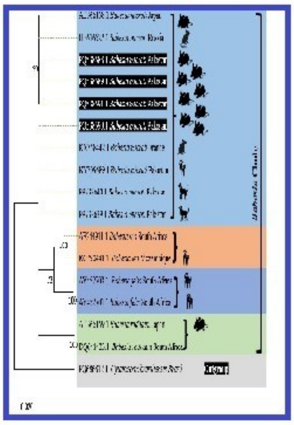

Supplement: Supplementary file 1 [file VMS3-12-e71196-s002.docx]
