# Supplementary material for: Molecular Detection, Epidemiology and Genetic Diversity of Babesia microti Infecting Wild Rodents
Source: Vet Med Sci. 2026 Sep 3;12(5):e71196. doi: 10.1002/vms3.71196 (PMC13542696; doi:10.1002/vms3.71196)
Supplement: Supplementary file 1 [file VMS3-12-e71196-s001.docx]

**Supplementary Fig. 1.** Overall comparison of body weight between *Babesia microti* infected and uninfected wild rodents’ species captured from seven districts of Pakistan during present investigation. Data is presented as mean ± standard error of mean. P-value represents the outcome of a sample t-test.
